# Efficacy and safety of bispecific antibodies versus other antitumor therapies in solid tumors: a systematic review and meta-analysis

**DOI:** 10.3389/fimmu.2026.1931637

**Published:** 2026-08-31

**Authors:** Yici Yan, Weidong Gao, Zhenzheng Zhu, Leyi Zheng, Yujie Lu, Leitao Sun, Guanjun Jiang

**Affiliations:** 1 The First Affiliated Hospital of Zhejiang Chinese Medical University (Zhejiang Provincial Hospital of Chinese Medicine), Hangzhou, China; 2 Key Laboratory of Neuropharmacology and Translational Medicine of Zhejiang Province, School of Pharmaceutical Sciences, Zhejiang Chinese Medical University, Hangzhou, China; 3 Longyou Hospital of Chinese Medicine, Quzhou, China

**Keywords:** bispecific antibody, efficacy, meta-analysis, safety, solid tumor

## Abstract

**Background:**

Bispecific antibodies (BsAbs) have emerged as a promising strategy for solid tumor treatment, yet their comparative efficacy and safety versus other antitumor therapies remain unclear.

**Materials and methods:**

Literature was systematically searched in PubMed, Embase, Cochrane Library, and Scopus from inception up to August 2026. American Society of Clinical Oncology (ASCO), European Society for Medical Oncology (ESMO) and clinicaltrials.gov were also checked. Progression-free survival (PFS), overall survival (OS), overall response rate (ORR), and adverse events (AEs) were used to assess efficacy and safety. Publication bias was assessed using funnel plots. Heterogeneity was evaluated using subgroup, meta-regression and sensitivity analyses. The protocol was preregistered in the International Prospective Register of Systematic Reviews (CRD420261359397).

**Results:**

A total of 9 eligible studies involving 3,505 patients were included. Compared with other antitumor therapies, BsAbs demonstrated significant improvements in PFS (hazard ratio [HR]: 0.76, 95% confidence interval [CI]: 0.61-0.94, p=0.011) and OS (HR: 0.78, 95% CI: 0.63-0.95, p=0.016). ORR showed a borderline effect (Risk Ratio [RR]: 1.20, 95% CI: 1.00-1.44, p=0.046). Subgroup analyses suggested potential benefits in selected populations, particularly among patients treated with T-cell engaging BsAbs or tumor microenvironment/angiogenesis-modulating BsAbs, patients aged <65 years, and patients with non-small cell lung cancer (NSCLC). Regarding safety, renal and vascular toxicities, including proteinuria, peripheral edema, and hypertension, as well as immune-related toxicities such as cytokine release syndrome and rash, were more frequently observed in the BsAb group. Other increased adverse events included anemia, pain in extremity, decreased appetite, and vomiting.

**Conclusion:**

BsAb-based regimens significantly improve PFS and OS compared with non-BsAb antitumor therapies in patients with solid tumors. Although the overall AE profile appeared manageable, renal and vascular toxicities and immune-related/inflammatory toxicities warrant particular attention.

**Systematic review registration:**

https://www.crd.york.ac.uk/PROSPERO/, identifier CRD420261359397.

## 1 Introduction

The landscape of systemic therapy for solid tumors has broadened considerably. Where conventional chemotherapy once dominated, treatment now encompasses targeted agents, immune checkpoint inhibitors (ICI), antiangiogenic drugs, and monoclonal antibodies (1–3). These strategies have improved clinical outcomes across multiple tumor types (4, 5). However, several limitations remain. Agents directed at a single target often encounter pathway compensation, intratumoral heterogeneity, and acquired resistance over time. Combination strategies, on the other hand, tend to bring added toxicity and introduce greater complexity into treatment decisions (6–8). In this context, regimens that achieve coordinated antitumor activity without a commensurate increase in adverse effects remain a worthwhile pursuit.

Bispecific Antibodies (BsAbs) are antibody-based therapeutics capable of engaging two distinct antigens or epitopes within a single molecular construct (9). Depending on their targets, this design allows them to exert antitumor effects through mechanisms such as dual pathway inhibition, immune-cell redirection, immune checkpoint modulation, or regulation of the tumor microenvironment (10, 11). Recent clinical development of BsAbs in solid tumors has expanded across diverse targets, including human epidermal growth factor receptor 2 (HER2), delta-like ligand 3 (DLL3), claudin 18.2 (CLDN18.2), programmed death 1 (PD-1)/vascular endothelial growth factor (VEGF), and epidermal growth factor receptor (EGFR)/mesenchymal-epithelial transition factor (MET) (12, 13). This diversity provides a biological rationale for their use in solid tumors, but also indicates that BsAbs should not be regarded as a homogeneous therapeutic class. Recent clinical studies further illustrate this expanding and heterogeneous therapeutic landscape. In a phase II study, KN026 plus docetaxel achieved an overall response rate (ORR) of 76.4% in HER2-positive recurrent or metastatic breast cancer (14). In the phase III DeLLphi-304 trial, tarlatamab prolonged overall survival (OS) compared with chemotherapy in small-cell lung cancer (HR: 0.60; 95% CI: 0.47-0.77; p<0.001) (15). In the COMPASSION-16 trial, cadonilimab improved OS in cervical cancer (hazard ratio [HR]: 0.64; 95% confidence interval [CI]: 0.48-0.86; p=0.001) (16). However, in the MEHGAN study of recurrent or metastatic squamous cell carcinoma of the head and neck (SCCHN), duligotuzumab did not improve OS compared with cetuximab (7.2 vs. 8.7 months; HR: 1.15; 90% CI: 0.81-1.63) (17). These findings suggest that the value of BsAbs cannot be judged only by comparisons with placebo or weak background treatment.

For clinical decision-making, the more relevant question is whether BsAbs provide additional efficacy over existing non-BsAb antitumor therapies with an acceptable safety profile. Although previous systematic reviews have provided valuable insights into the activity of BsAbs in solid tumors, several limitations remain. Nejadghaderi et al. (18) included heterogeneous comparative designs, combining studies evaluating the added value of BsAbs to existing regimens with those comparing BsAbs against alternative antitumor therapies. Yan et al. (19) focused on BsAbs combined with chemotherapy versus chemotherapy alone, assessing the incremental benefit of BsAbs rather than their efficacy compared with other established antitumor therapies. Therefore, a meta-analysis of randomized controlled trials (RCTs) was conducted to compare BsAb-based treatment with non-BsAb antitumor therapy in patients with solid tumors.

## 2 Methods

### 2.1 Literature search strategy

The present meta-analysis followed the reporting principles of the Preferred Reporting Items for Systematic Reviews and Meta-Analyses (PRISMA) statement. The protocol had been registered prospectively in the International Prospective Register of Systematic Reviews under registration number CRD420261359397. A comprehensive literature search was conducted in Embase, PubMed, the Cochrane Library, and Scopus, covering all available records from database inception to August 2026. The core search strategy combined the terms “bispecific antibody” AND “neoplasm”. American Society of Clinical Oncology (ASCO), European Society for Medical Oncology (ESMO) were also checked and the detailed search strategy is provided in **Supplementary Table 1**. Reference lists of eligible articles were also checked to minimize the possibility of missing pertinent trials.

### 2.2 Inclusion and exclusion criteria

Studies were included when they met the following requirements: (1) enrolling patients with solid tumors; (2) comparing a BsAb-containing regimen with non-BsAb antitumor therapy; (3) reporting at least one time-to-event efficacy outcome, including progression-free survival (PFS) or OS; and (4) RCTs.

Studies were excluded when they met any of the following conditions: (1) not providing extractable efficacy data, particularly hazard ratios (HRs) with corresponding 95% confidence intervals (CIs) for PFS or OS; (2) lacking full-text availability; (3) using placebo alone as the control intervention rather than an active non-BsAb antitumor therapy.

Study selection was conducted by two independent investigators. Any discrepancies between the two investigators were first discussed and resolved by consensus. If agreement could not be reached, a third investigator was involved to make the final decision.

### 2.3 Data extraction

Two investigators extracted data from eligible studies using a predefined data collection form, and the extracted information was cross-checked before analysis. The following data were collected: (1) study-level characteristics: first author, publication year, trial phase, cancer type, study region, treatment line, follow-up duration, sample size, experimental regimen, control regimen, and therapeutic target; (2) efficacy outcomes: HRs with 95% CIs for PFS and OS, and ORR; and (3) safety outcomes: all-grade adverse events (AEs), grade 3 or higher AEs.

### 2.4 Quality assessment

Two reviewers independently evaluated the methodological quality of the included RCTs using the Cochrane risk-of-bias tool (RoB 1.0). The risk-of-bias assessments were performed using Review Manager version 5.4. The certainty of evidence for each outcome was assessed using the Grading of Recommendations Assessment, Development, and Evaluation (GRADE) framework. Any inconsistency in judgment was resolved by discussion or adjudication by a third reviewer.

### 2.5 Statistical analysis

Stata version 18.0 was used for all statistical analyses. HRs with 95% CIs were used as the effect measure for PFS and OS. An HR below 1 was interpreted as favoring the BsAb-containing regimen, whereas an HR above 1 indicated a direction favoring the control treatment. Dichotomous outcomes, including ORR and safety endpoints, were summarized using relative risks (RRs) with corresponding 95% CIs. For ORR, an RR greater than 1 represented a higher response rate in the BsAb group; for AE-related outcomes, it indicated a higher incidence of toxicity in the same group. Heterogeneity across studies was evaluated using Cochran’s Q test and further quantified by the I² statistic. I² values of less than 30%, 30% - 60%, and greater than 60% were interpreted as low, moderate, and substantial heterogeneity, respectively. Given the expected differences in tumor types, treatment regimens, drug targets, and comparator therapies across the included trials, the main pooled estimates were generated using a random-effects model. To investigate the potential sources of substantial heterogeneity and to assess whether the treatment effect varies across different clinical settings, we conducted prespecified subgroup analyses stratified by clinically relevant characteristics, including BsAb format, BsAb mechanism, cancer type, and comparator therapy. Additional exploratory subgroup analyses were performed according to patient and disease characteristics when sufficient data were available. Meta-regression analyses were also performed to explore potential sources of heterogeneity. Sensitivity analyses were performed by sequentially omitting individual studies to examine the robustness of the pooled estimates. Potential publication bias was assessed using funnel plots. All statistical tests were two-sided, and a P value<0.05 was considered statistically significant.

## 3 Results

### 3.1 Literature search results

**Figure 1:**
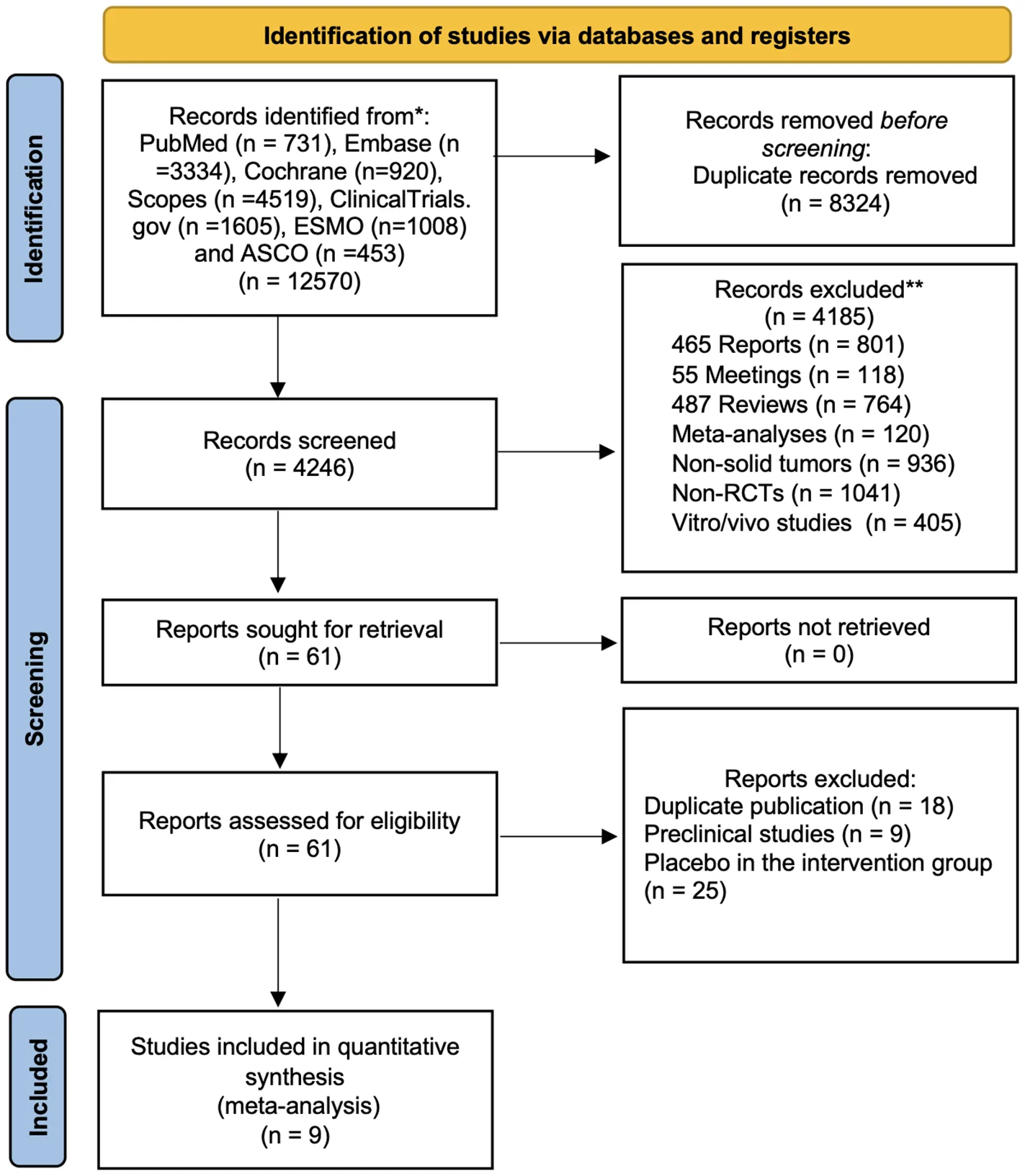
Flowchart of the study selection.

The database search yielded 12,570 records. After 8324 duplicates were removed, 4,246 records were screened by title and abstract. During this stage, 4,185 records were excluded for not meeting the eligibility criteria. The remaining records were further checked for relevance, and 61 reports were retrieved for full-text evaluation. All full texts were successfully obtained. Following full-text review, 52 reports were excluded, including 18 duplicate publications, 9 preclinical studies, and 25 studies in which placebo was used as the control intervention rather than an active non-BsAb antitumor therapy. Finally, 9 studies met the eligibility criteria and were included in the quantitative synthesis. The detailed study selection process is shown in **Figure 1**.

### 3.2 Basic characteristics of included studies

Nine studies comprising 3,505 patients were included in the present meta-analysis (15, 17, 20–26). The publication years ranged from 2016 to 2025, and the trials were mainly conducted in China, the United States, and Europe. Most studies investigated first-line treatment, while several studies evaluated later-line treatment settings. The median follow-up ranged from 8.7 to 43.3 months. The included studies covered multiple solid tumors, including colorectal cancer (CRC), NSCLC, SCCHN, uveal melanoma, and nasopharyngeal carcinoma. The BsAb-based regimens in the experimental groups included vanucizumab, ivonescimab, duligotuzumab, tebentafusp, tarlatamab, amivantamab plus lazertinib, and iza-bren. The control regimens included bevacizumab, tislelizumab, cetuximab, pembrolizumab, ipilimumab, dacarbazine, osimertinib, and chemotherapy-based treatment. The detailed characteristics are provided in **Table 1** and **Supplementary Table 2**.

**Table 1:** Characteristic of included studies.

Author, year	Location	Line	Cancer type	Follow-up, months	Sample size	Intervention group Control group	No. of patients	Median PFS, months	Median OS, months
Bendell JC, 2020 (20)	China	1	CRC	17.6	189	I: Vanucizumab	94	7.2	NE
						C: Bevacizumab	95	7.1	NE
Chen Z, 2025 (21)	China	1	NSCLC	10.3	532	I: Ivonescimab	266	11.1	NE
						C: Tislelizumab	266	6.9	NE
Fayette J, 2016 (17)	NE	≥1	SCCHN	NE	121	I: Duligotuzumab	59	4.2	7.2
						C: Cetuximab	62	4.0	8.7
Hassel JC, 2023 (22)	United States and Europe	1	Uveal Melanoma	43.3	378	I: Tebentafusp	252	3.4	21.6
						C: Pembrolizumab, Ipilimumab, or Dacarbazine	126	2.9	16.9
Hill AG, 2018 (23)	NE	NE	CRC	NE	134	I: Duligotuzumab	68	5.4	14.0
						C: Cetuximab	66	5.6	12.4
Mountzios G, 2025 (15)	NE	NE	NSCLC	37.8	509	I: Tarlatamab	254	5.3	13.6
						C: Chemotherapy	255	4.3	8.3
Xiong A, 2025 (24)	China	1	NSCLC	8.7	398	I: Ivonescimab	198	11.1	NE
						C: Pembrolizumab	200	5.8	NE
Yang JC, 2025 (25)	NE	NE	NSCLC	37.8	858	I: Amivantamab- Lazertinib	429	25.4	NE
						C: Osimertinib	429	22.2	36.7
Yang Y, 2025 (26)	China	≥1	Nasopharyngeal Carcinoma	NE	386	I: Iza-bren	191	8.38	7.66
						C: Chemotherapy	195	4.34	7.1

NE, not estimated; CRC, colorectal cancer; NSCLC, non-small cell lung cancer; SCCHN, Squamous Cell Carcinoma of the Head and Neck; I, intervention group; C, control group; No, number; PFS, progression-free survival; OS, overall survival.

### 3.3 Efficacy

**Figure 2:**
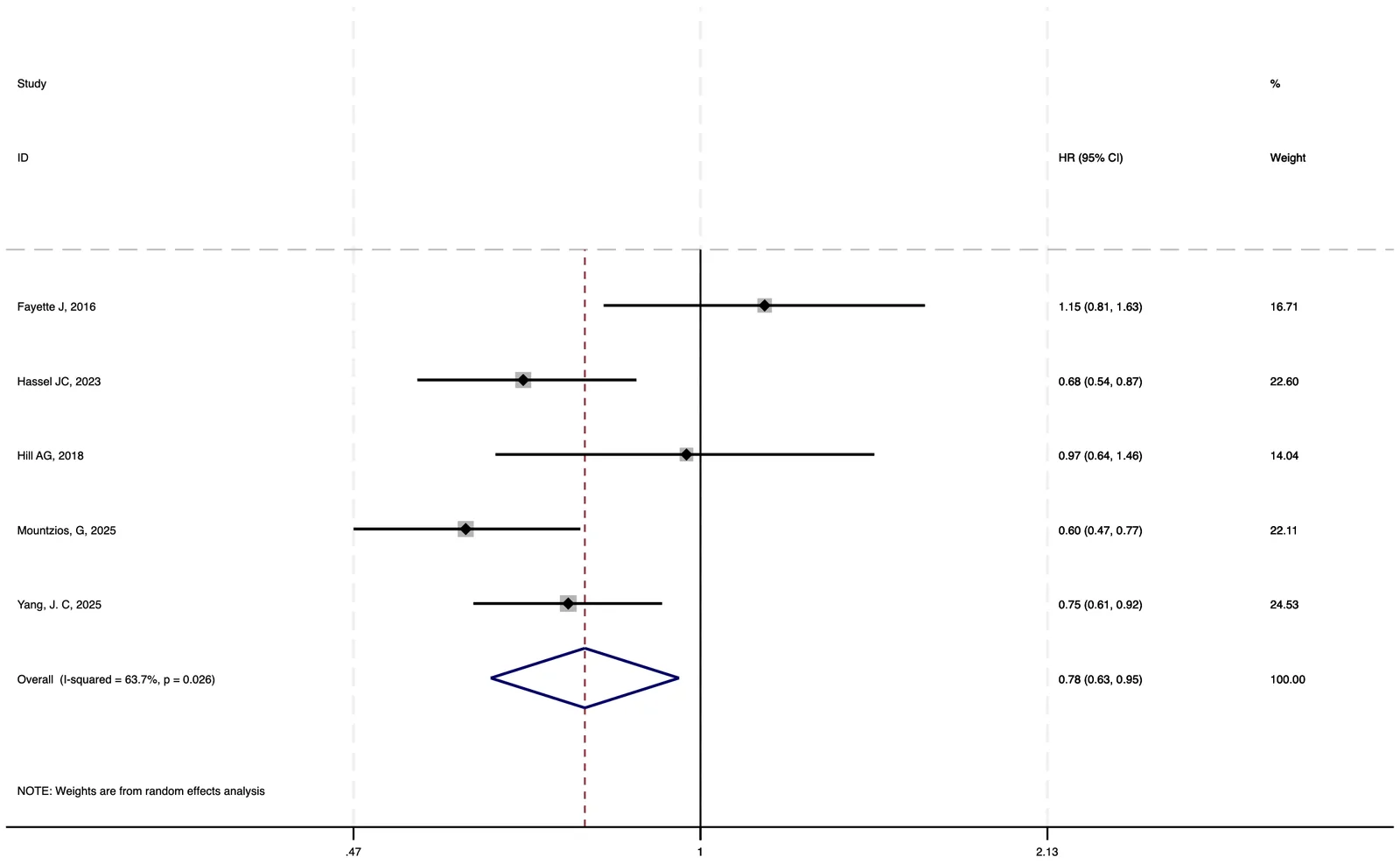
Forest plot of the meta-analysis on PFS.

**Figure 3:**
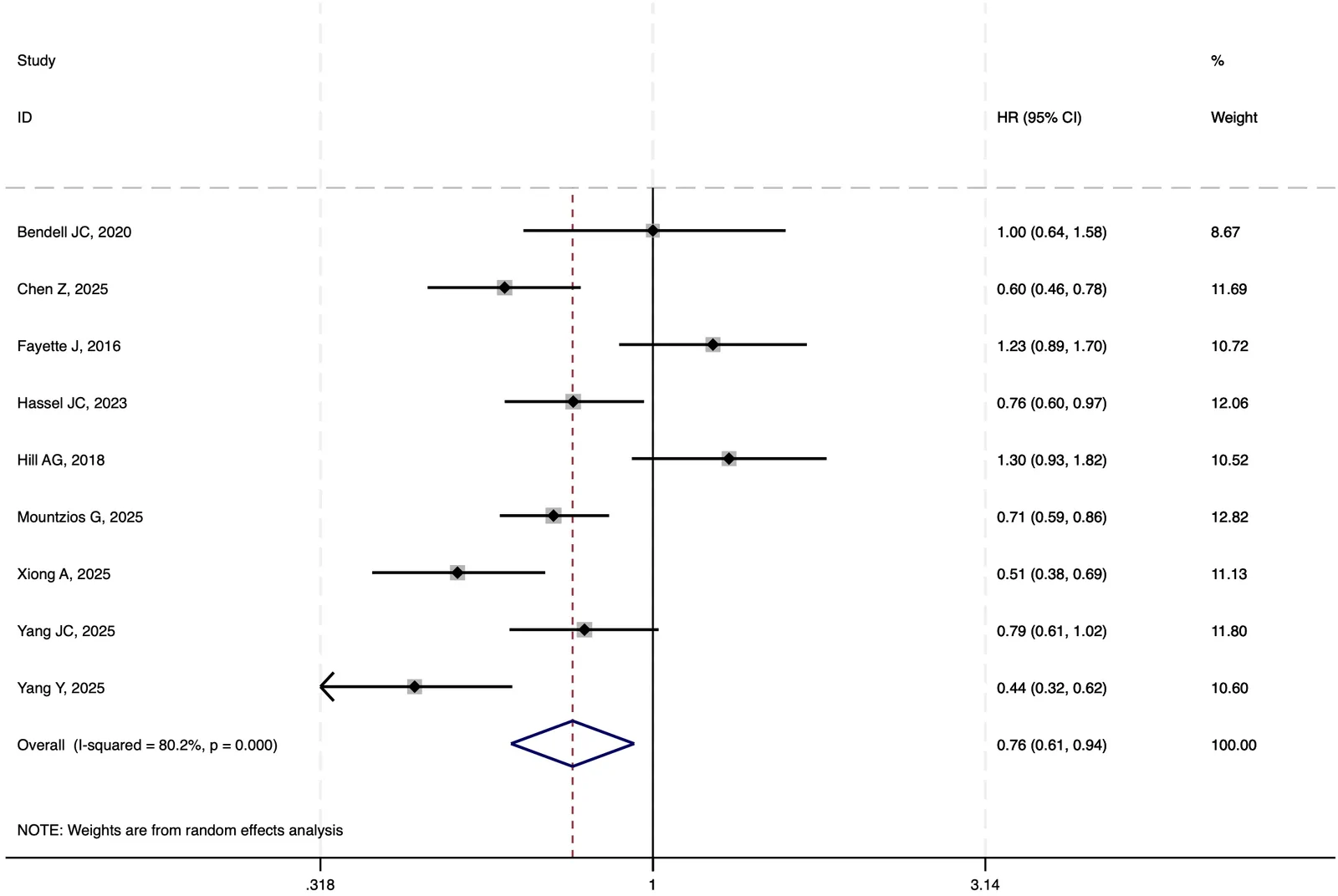
Forest plot of the meta-analysis on OS.

**Figure 4:**
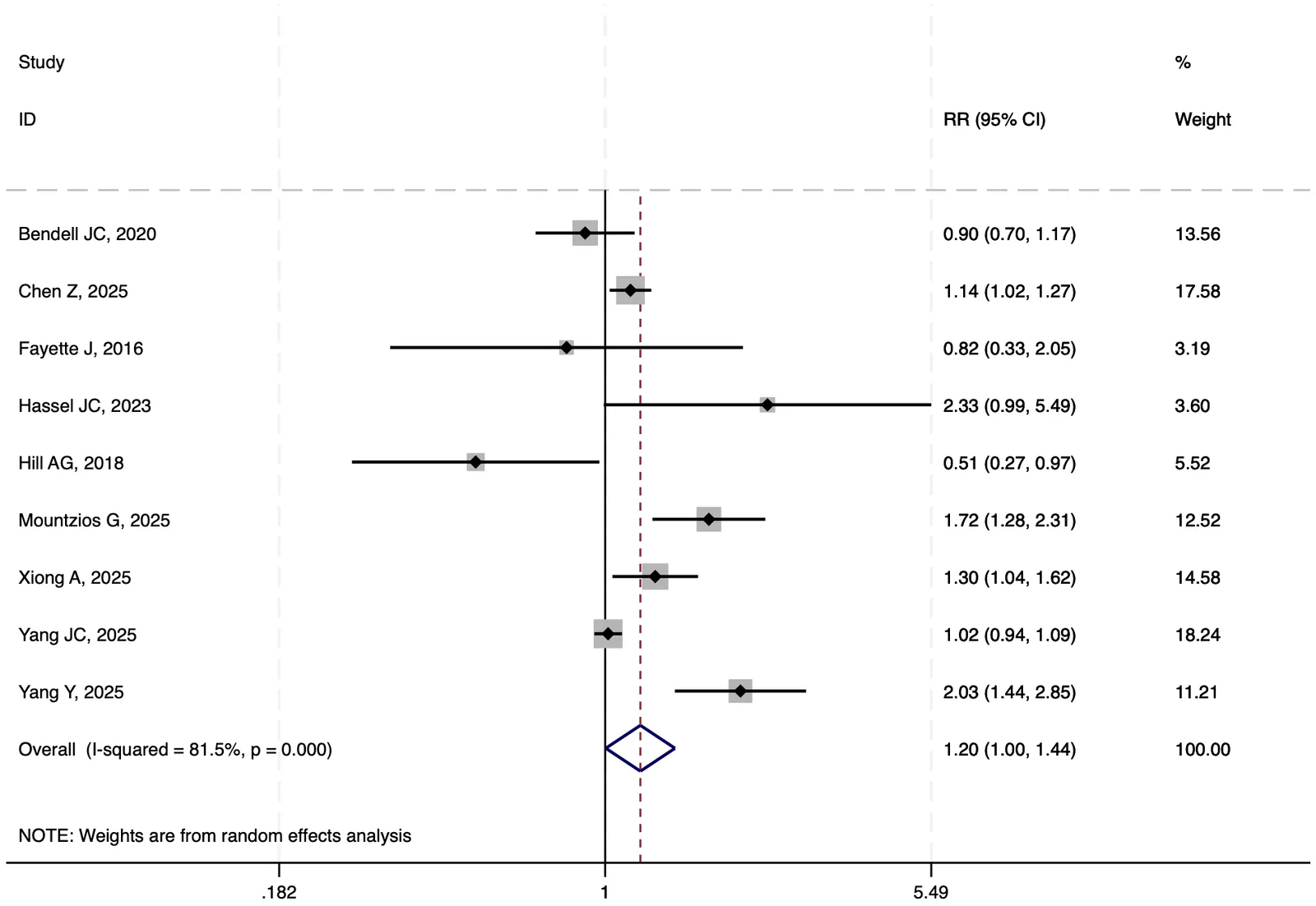
Forest plot of the meta-analysis on ORR.

All 9 included studies reported PFS. The HR for PFS was 0.76 (95% CI: 0.61-0.94, p=0.011, **Figure 2**). Substantial heterogeneity was observed across the included studies (I²=80.2%). OS was reported in 5 studies. The HR for OS was 0.78 (95% CI: 0.63-0.95, p=0.016, **Figure 3**). The heterogeneity among studies was moderate to substantial (I²=63.7%). All 9 included studies reported ORR. The RR for ORR was 1.20 (95% CI: 1.00-1.44, p=0.046, **Figure 4**), with considerable heterogeneity across studies (I²=81.5%).

### 3.4 Subgroup analysis

Prespecified subgroup analyses were performed according to BsAb format, mechanism, cancer type, and comparator therapy (**Table 2**). For BsAb format, non-IgG-like BsAbs showed a significant improvement in PFS (HR: 0.73; 95% CI: 0.63-0.85; p<0.01), whereas IgG-like BsAbs did not show a statistically significant difference (p=0.10). For BsAb mechanism, T-cell engaging BsAbs demonstrated significant improvements in PFS (HR: 0.73; 95% CI: 0.63-0.85; p<0.01) and OS (HR: 0.64; 95% CI: 0.54-0.76; p<0.01). Tumor microenvironment-modulating BsAbs were associated with improved PFS (HR: 0.65; 95% CI: 0.47-0.90; p<0.01), whereas tumor signaling pathway-targeting BsAbs did not show statistically significant differences in PFS or OS (p=0.53 and p=0.51, respectively). For comparator therapy, BsAb-based regimens showed significant PFS improvements compared with chemotherapy-based comparators (HR: 0.57; 95% CI: 0.36-0.91; p=0.02) and immunotherapy-based comparators (HR: 0.62; 95% CI: 0.50-0.78; p<0.01). No statistically significant difference was observed in studies using targeted therapy-based comparators (p=0.72). For cancer type, significant PFS improvement was observed in patients with NSCLC (HR: 0.65; 95% CI: 0.55-0.78; p<0.01), whereas no statistically significant difference was observed in patients with CRC (p=0.22).

**Table 2:** Pre-specified subgroup analysis of PFS and OS.

Subgroup	PFS					OS				
	No. of studies	HR (95% CI)	I^2^ (%)	p	Meta- regression p	No. of studies	HR (95% CI)	I^2^ (%)	p	Meta- regression p
BsAb format										
Non-IgG-like	2	0.73 (0.63, 0.85)	0.0	<0.01	0.88	2	0.74 (0.46, 1.18)	73.9	0.21	0.14
IgG-like	7	0.77 (0.57, 1.05)	85.1	0.10		3	0.81 (0.62, 1.05)	67.5	0.12	
BsAbs mechanism										
T-cell engaging BsAbs	2	0.73 (0.63, 0.85)	0.0	<0.01	0.55	2	0.64 (0.54, 0.76)	0.0	<0.01	0.15
Tumor microenvironment-modulating BsAbs	3	0.65 (0.47, 0.90)	66.6	<0.01		NA	NA	NA	NA	
Tumor signaling pathway-targetin BsAbs	4	0.86 (0.54, 1.37)	88.8	0.53		3	0.91 (0.69, 1.20)	57.3	0.51	
Cancer type										
CRC	2	1.18 (0.90, 1.55)	0.0	0.22	0.83	NA	NA	NA	NA	NA
NSCLC	4	0.65 (0.55, 0.78)	48.4	<0.01		NA	NA	NA	NA	
Comparator therapy										
Chemotherapy	2	0.57 (0.36, 0.91)	83.5	0.02	0.03	NA	NA	NA	NA	NA
Immunotherapy	3	0.62 (0.50, 0.78)	54.5	<0.01		NA	NA	NA	NA	
Targeted therapy	4	1.05 (0.81, 1.35)	57.7	0.72		NA	NA	NA	NA	

CRC, colorectal Cancer; NSCLC, non-small cell lung cancer; No, number; PFS, progression-free survival; HR, hazard ratio; OS, overall survival; NA, not available.

For exploratory subgroup analyses (**Supplementary Table 3**), improvements in PFS were observed in patients aged <65 years (HR: 0.56; 95% CI: 0.37-0.84; p=0.01) and ≥65 years (HR: 0.67; 95% CI: 0.52-0.88; p<0.01). Similar benefits were observed in patients with brain metastasis (HR: 0.59; 95% CI: 0.45-0.77; p<0.01) and without brain metastasis (HR: 0.68; 95% CI: 0.52-0.89; p<0.01). PFS improvements were also observed across ECOG PS 0 (HR: 0.35; 95% CI: 0.18-0.68; p<0.01) and ECOG PS 1 (HR: 0.57; 95% CI: 0.48-0.69; p<0.01), as well as in patients with liver metastasis (HR: 0.61; 95% CI: 0.44-0.85; p<0.01) and without liver metastasis (HR: 0.57; 95% CI: 0.45-0.72; p<0.01). PD-L1 expression subgroups also showed significant PFS benefits in both the 1%-49% subgroup (HR: 0.56; 95% CI: 0.40-0.78; p<0.01) and ≥50% subgroup (HR: 0.58; 95% CI: 0.42-0.80; p<0.01). Sex-based analyses demonstrated consistent PFS benefits in both female (HR: 0.55; 95% CI: 0.41-0.75; p<0.01) and male patients (HR: 0.58; 95% CI: 0.46-0.74; p<0.01). For OS, exploratory analyses showed benefits in patients aged <65 years (HR: 0.56; 95% CI: 0.46-0.67; p<0.01), those with brain metastasis (HR: 0.56; 95% CI: 0.38-0.82; p<0.01), Asian patients (HR: 0.63; 95% CI: 0.43-0.91; p=0.02), and non-Asian patients (HR: 0.74; 95% CI: 0.58-0.95; p=0.02). Sex-based analyses showed significant OS benefits in both female (HR: 0.65; 95% CI: 0.50-0.86; p<0.01) and male patients (HR: 0.73; 95% CI: 0.61-0.87; p<0.01), whereas no statistically significant difference was observed in patients aged ≥65 years (p=0.34).

### 3.5 Safety

Treatment-related AEs are shown in **Table 3**. For digestive system AEs, all-grade decreased appetite (RR: 1.32; 95% CI: 1.05-1.66; p=0.019) and vomiting (RR: 1.52; 95% CI: 1.00-2.30; p=0.049) were more frequently reported in the BsAb group. No statistically significant differences were observed for all-grade diarrhea (p=0.360) or nausea (p=0.629). For grade ≥3 digestive system AEs, decreased appetite (p=0.960), diarrhea (p=0.062), nausea (p=0.120), and vomiting (p=0.793) showed no statistically significant differences between groups.

**Table 3:** Treatment-related adverse events in this meta-analysis.

Adverse events		RR (95% CI)			
		All grade	P value	Grade≥3	P value
Digestive system	Decreased appetite	1.32 (1.05, 1.66)	0.019	1.02 (0.45, 2.34)	0.960
	Diarrhea	1.21 (0.81, 1.81)	0.360	1.78 (0.97, 3.25)	0.062
	Nausea	1.11 (0.73, 1.67)	0.629	2.67 (0.77, 9.24)	0.120
	Vomiting	1.52 (1.00, 2.30)	0.049	1.19 (0.33, 4.31)	0.793
Hematological system	Anemia	1.06 (0.76, 1.49)	0.724	3.12 (1.27, 7.62)	0.013
	Leukopenia	0.55 (0.35, 0.85)	0.007	0.43 (0.06, 2.94)	0.388
	Neutropenia	0.64 (0.33, 1.26)	0.199	0.56 (0.25, 1.24)	0.155
Renal and vascular events	Hypertension	6.87 (2.86, 16.51)	<0.001	9.93 (1.89, 52.09)	0.007
	Proteinuria	2.45 (1.64, 3.66)	<0.001	10.88 (1.41, 84.18)	0.022
	P-Edema	4.49 (2.25, 8.94)	<0.001	8.13 (1.02, 64.74)	0.048
Immune-related and inflammatory event	CRS	61.24 (16.82, 222.96)	<0.001	2.29 (0.11, 47.22)	0.593
	Pyrexia	3.69 (1.08, 12.66)	0.038	10.51 (0.63, 176.85)	0.102
	Hypothyroidism	1.18 (0.77, 1.80)	0.458	NA	NA
	Rash	1.35 (0.81, 2.24)	0.244	7.86 (1.13, 54.47)	0.037
Others	Alopecia	1.10 (0.83, 1.47)	0.511	0.31(0.01, 7.56)	0.475
	Arthralgia	1.02 (0.52, 2.01)	0.948	1.07 (0.08, 14.66)	0.961
	Fatigue	1.24 (0.98, 1.56)	0.078	0.77 (0.44, 1.34)	0.349
	IRR	19.47 (0.00, 2.59e+07)	0.680	3.04 (0.01, 1361.69)	0.721
	Pain in extremity	1.92 (1.20, 3.08)	0.007	3.02 (0.61, 14.90)	0.175

P-Edema, peripheral edema; CRS, Cytokine release syndrome; IRR, Infusion-related reaction; RR, relative risk; NA, not available.

For hematological AEs, all-grade leukopenia was less frequently reported in the BsAb group than in the control group (RR: 0.55; 95% CI: 0.35-0.85; p=0.007). All-grade anemia (p=0.724) and neutropenia (p=0.199) were not statistically significant. Among grade ≥3 hematological AEs, anemia was more frequent in the BsAb group (RR: 3.12; 95% CI: 1.27-7.62; p=0.013), whereas leukopenia (p=0.388) and neutropenia (p=0.155) showed no statistically significant differences.

For renal and vascular AEs, all-grade hypertension (RR: 6.87; 95% CI: 2.86-16.51; p<0.001), proteinuria (RR: 2.45; 95% CI: 1.64-3.66; p<0.001) and peripheral edema (RR: 4.49; 95% CI: 2.25-8.94; p<0.001) were more frequently reported in the BsAb group. Grade ≥3 hypertension (RR: 9.93; 95% CI: 1.89-52.09; p=0.007), proteinuria (RR: 10.88; 95% CI: 1.41-84.18; p=0.022) and peripheral edema (RR: 8.13; 95% CI: 1.02-64.74; p=0.048) were also more frequent in the BsAb group.

For immune-related or inflammatory AEs, all-grade cytokine release syndrome (CRS) (RR: 61.24; 95% CI: 16.82-222.96; p<0.001) and pyrexia (RR: 3.69; 95% CI: 1.08-12.66; p=0.038) were more frequently reported in the BsAb group. No statistically significant differences were observed for all-grade hypothyroidism (p=0.458) or rash (p=0.244). For grade ≥3 immune-related or inflammatory AEs, rash was more frequent in the BsAb group (RR: 7.86; 95% CI: 1.13-54.47; p=0.037). Grade ≥3 CRS (p=0.593) and pyrexia (p=0.102) showed no statistically significant differences between groups.

Among other AEs, all-grade pain in extremity was more frequently reported in the BsAb group (RR: 1.92; 95% CI: 1.20-3.08; p=0.007). No statistically significant differences were observed for all-grade alopecia (p=0.511), arthralgia (p=0.948), fatigue (p=0.078), or infusion-related reaction (p=0.680). For grade ≥3 other AEs, alopecia (p=0.475), arthralgia (p=0.961), fatigue (p=0.349), pain in extremity (p=0.175), and infusion-related reaction (p=0.721) showed no statistically significant differences between groups.

### 3.6 Quality assessment

The risk-of-bias assessment for individual trials is detailed in **Supplementary Figures 1** and **S2**. In total, 6 trials were classified as having high reliability with a low risk of bias, while three were deemed to have moderate reliability. Based on the GRADE assessment of the evidence, the certainty of evidence for PFS and OS was rated as moderate and the certainty of evidence for ORR was rated as low (**Supplementary Table 4**).

### 3.7 Publication bias and sensitivity analysis

Publication bias was explored using funnel plots. The funnel plot for PFS appeared generally symmetrical, suggesting no obvious evidence of publication bias (**Supplementary Figure 3**). The funnel plots for OS (**Supplementary Figure 4**) and ORR (**Supplementary Figure 5**) showed mild asymmetry, suggesting that publication bias could not be completely ruled out for these outcomes.

Sensitivity analysis was conducted by sequentially omitting each included study. The pooled estimates remained stable after individual study removal, suggesting that no single study substantially affected the overall results.

## 4 Discussion

As an emerging class of antitumor agents, BsAbs have been increasingly investigated in solid tumors (27), although previous evidence has mainly confirmed their activity in placebo-controlled or add-on treatment settings (28–30). In this study, additional clinical value of BsAbs over active non-BsAb antitumor therapies was evaluated through pooled analysis of 3,505 patients from 9 RCTs. The results showed statistically significant effects on PFS and OS, suggesting that BsAbs may offer efficacy beyond standard or routine treatments in selected solid tumors. This finding is in line with the increasing incorporation of selected BsAbs into major clinical guidelines. Notable examples include amivantamab-based regimens for EGFR-mutated NSCLC in the National Comprehensive Cancer Network (NCCN) guidelines (31), as well as tebentafusp for HLA-A*02:01-positive metastatic uveal melanoma in both NCCN and ASCO guidelines (32, 33). However, many BsAbs remain outside guideline-based recommendations, and the comparative value of BsAbs versus existing non-BsAb therapies has not been fully clarified. Therefore, our study provides a more clinically relevant assessment of BsAbs by focusing on active-comparator RCTs rather than placebo-based evidence alone.

As a supplementary endpoint reflecting tumor response, ORR was also analyzed in this study. The pooled result showed a borderline statistical effect, with an RR of 1.20 and a 95% CI reaching the null line. However, the subsequent subgroup analyses revealed that the overall borderline estimate did not uniformly apply across all patient populations. Notably, BsAbs demonstrated substantial tumor-shrinking activity in several specific subgroups in our study. ORR improvements were observed in patients treated with non-IgG-like BsAb formats (RR: 1.77; 95% CI 1.34-2.35, **Supplementary Table 5**) or T-cell-engaging BsAbs (RR: 1.77; 95% CI 1.34-2.35), and in those with NSCLC (RR: 1.22; 95% CI 1.02-1.45). These observations indicate that the modest overall estimate may therefore reflect the dilution of pronounced effects in responsive populations by the inclusion of broader, less responsive patient cohorts. In this context, the borderline overall ORR does not necessarily contradict the observed PFS and OS benefits. Rather, the diversity of mechanisms among different BsAbs may help explain this discrepancy. For instance, T-cell-engaging BsAbs may induce immune-mediated tumor control, whereas dual-target antibodies may exert their effects through pathway inhibition, antiangiogenic activity, or modulation of the tumor microenvironment (34, 35). These mechanisms may take longer to manifest their full therapeutic effect and may therefore translate into disease stabilization or prolonged survival without necessarily producing a marked increase in radiographic response rate in the overall population (10). Nevertheless, the substantial ORR benefit observed in selected subgroups supports the potential role of BsAbs as effective tumor-shrinking agents in appropriately selected patients. In clinical practice, for patients who require prompt tumor volume reduction, such as those considered for neoadjuvant therapy or those with tumors prone to compression, obstruction, or other local complications, specific BsAb formats or mechanisms may offer a viable therapeutic option. Overall, ORR should be interpreted as a complementary outcome rather than the sole indicator of BsAb efficacy, and the subgroup findings highlight the importance of patient selection and biomarker-driven strategies in optimizing BsAb therapy.

The differential efficacy patterns observed across BsAb subgroups may be closely related to their distinct molecular mechanisms of action, including immune-cell recruitment, tumor microenvironment modulation, and oncogenic pathway blockade. T-cell-engaging BsAbs represent a mechanistically distinct class that simultaneously binds to tumor-associated antigens and CD3 subunits on T cells, leading to the recruitment of T cells to the tumor, followed by T-cell activation, degranulation, and tumor cell elimination (34). This immune-redirecting mechanism bypasses conventional MHC class I-restricted antigen presentation and directly activates cytotoxic T-cell responses, allowing tumor-cell elimination independent of endogenous T-cell receptor recognition (36). The favorable efficacy signals observed for T-cell-engaging BsAbs in our analysis are consistent with this mechanism-driven therapeutic rationale. Corroborating our findings, the DeLLphi-300 trial showed that tarlatamab, a DLL3-targeting bispecific T-cell engager, exhibited durable antitumor activity in patients with previously treated small-cell lung cancer, with an ORR of 23.4%, a median PFS of 3.7 months, and a median OS of 13.2 months (37). Tumor microenvironment-modulating BsAbs represent another biologically distinct strategy. In our subgroup analysis, this category showed PFS improvement, which may be related to their ability to simultaneously regulate immune suppression and tumor-associated vascular abnormalities (38). Among these approaches, PD-1/VEGF dual-targeting BsAbs integrate immune checkpoint inhibition with angiogenesis blockade, potentially enhancing antitumor immunity through both T-cell activation and vascular normalization. Clinical data further support this concept, as JS207, a PD-1/VEGF-A bispecific antibody, has achieved objective response rates of 58.1% in PD-L1-positive NSCLC and 66.7% in hepatocellular carcinoma, with manageable safety profiles (39). Tumor signaling pathway-targeting BsAbs, in contrast, showed a non-significant PFS estimate in our analysis. These agents typically block oncogenic driver pathways such as EGFR, HER2, or MET through bispecific engagement of two receptors or a receptor and its ligand (10). While this approach has demonstrated efficacy in biomarker-selected populations, the lack of statistically significant benefit and the substantial variability observed likely reflect the dependence on specific genetic alterations, variable degrees of pathway redundancy, and the emergence of resistance mechanisms (40). Unlike T-cell-engaging or microenvironment-modulating BsAbs, which leverage the host immune system’s adaptive capacity, pathway-targeting BsAbs act directly on tumor cells and may be more susceptible to intrinsic or acquired resistance (41–43). This mechanistic distinction may explain the wider confidence intervals and greater variability in effect estimates observed for this category. Nevertheless, this pathway-directed strategy is not intrinsically ineffective. Rather, its efficacy is highly contingent on precise biomarker selection. Supporting this notion, in the phase 3 HERIZON-GEA trial, zanidatamab-containing regimens improved PFS compared with trastuzumab plus chemotherapy, with median PFS of 12.4 months versus 8.1 months (HR: 0.63-0.65, P<0.001) in a HER2-positive population (44). Thus, while population-level estimates for this category may appear diluted, the underlying biological rationale remains robust in genomically enriched subsets.

Subgroup analysis also showed a statistically significant PFS benefit in patients with NSCLC receiving BsAbs. This finding is supported by recent clinical evidence in NSCLC. In the HARMONi-A study, ivonescimab plus chemotherapy improved OS in patients with EGFR-variant nonsquamous NSCLC, with a median OS of 16.8 months versus 14.1 months in the control group (HR: 0.74; 95% CI: 0.58-0.95; p=0.02) (45). In the MARIPOSA-2 study, amivantamab-containing regimens also showed significant PFS improvement after progression on Osimertinib (29). The median PFS was 6.3 months with amivantamab-chemotherapy and 8.3 months with amivantamab-lazertinib-chemotherapy, compared with 4.2 months with chemotherapy alone. This may be partly explained by the disease biology and the current treatment framework of NSCLC. In clinical practice, NSCLC is among the solid tumors in which treatment selection is strongly shaped by molecular and immune biomarkers, such as EGFR mutation, ALK rearrangement, PD-L1 expression, and other molecular features (46, 47). More importantly, resistance after targeted therapy often involves bypass pathway activation, including MET amplification or other compensatory signaling mechanisms, while angiogenesis and immune escape also contribute to disease progression (48, 49). In this context, BsAbs designed to act on paired mechanisms may have particular relevance in selected NSCLC populations. For example, EGFR-MET- or PD-1-VEGF-directed BsAbs may simultaneously address a driver pathway and a resistance- or microenvironment-related process within one therapeutic construct (50–52). This biological rationale has been translated into clinical practice more rapidly in NSCLC. Consistently, NCCN guidelines have recommended amivantamab-based regimens for selected patients with EGFR-mutated advanced or metastatic NSCLC (31). However, the role of BsAbs in CRC has not yet been clearly positioned within established treatment algorithms, and major guidelines have not provided comparable BsAb-specific recommendations for CRC. Unlike NSCLC, where EGFR mutations and MET amplification provide clearly defined dual-targeting opportunities, the molecular framework of CRC is largely dominated by RAS/BRAF mutational status and mismatch repair proficiency, each of which directs treatment toward distinct, well-established monoclonal antibody-based strategies rather than bispecific approaches (53, 54). In RAS wild-type left-sided CRC, anti-EGFR monoclonal antibodies remain a cornerstone of therapy, while MSI-H tumors are effectively managed with PD-1 inhibitors (55). The therapeutic niches where a single BsAb could simultaneously address two clinically validated targets may have therefore been less apparent in CRC. Notably, only two studies contributed to this subgroup, which limits statistical power and makes the pooled estimate unstable. Therefore, the current CRC subgroup result should not be regarded as evidence of ineffectiveness, but rather as an indication that further biomarker-enriched and active-comparator trials are needed.

Notably, the pooled analyses revealed substantial between-study heterogeneity across all three efficacy endpoints, with I² values of 80.2% for PFS, 63.7% for OS, and 81.5% for ORR. To explore the potential sources of this heterogeneity, we performed pre-specified subgroup analyses stratified by BsAb format, mechanism of action, cancer type, and comparator therapy, followed by meta-regression analyses to formally test for effect modification (**Table 2**). Subgroup analyses stratified by biological characteristics showed varying degrees of residual heterogeneity across different categories, with heterogeneity reduced in some subgroups but remaining substantial in others, suggesting that molecular design and tumor type may partially contribute to the observed variability. Comparator-based subgroup analyses also demonstrated differences in residual heterogeneity across treatment settings, indicating that comparator selection may represent an important clinical factor influencing the magnitude and consistency of observed treatment effects. Exploratory meta-regression analyses were further performed to evaluate whether study-level characteristics were associated with treatment effect variation. For PFS, comparator therapy was significantly associated with effect estimates (p=0.03), whereas BsAb mechanism, format, and cancer type were not identified as significant moderators. Similarly, for ORR, comparator therapy was significantly associated with treatment effect variation (p=0.002), while no significant associations were observed for BsAb mechanism, format, or cancer type. These findings suggest that although biological differences among BsAb platforms may contribute to variations observed in subgroup analyses, the clinical context in which BsAbs are compared may represent a more consistent contributor to heterogeneity across studies. The lack of significant associations for mechanism-related variables in meta-regression does not exclude potential biological differences, but may reflect the limited number of available trials and insufficient statistical power. For OS, moderate heterogeneity was observed (I²=63.7%). However, the relatively smaller number of studies reporting OS outcomes limited the ability to identify potential modifiers. Neither BsAb format nor mechanism showed significant associations with OS effect estimates in meta-regression analyses, suggesting that additional evidence is required to clarify factors influencing long-term survival outcomes. In addition, sensitivity analyses were performed to evaluate whether specific treatment strategies influenced the pooled estimates. The Yang JC, 2025 trial evaluated an amivantamab-lazertinib combination regimen, whereas all other included studies evaluated BsAb monotherapy strategies. After excluding this study, the pooled estimates remained consistent with the primary analyses, suggesting that the overall efficacy findings were not substantially driven by combination therapy. Collectively, these analyses indicate that the efficacy of BsAbs should not be interpreted as a uniform class effect. Instead, treatment effects appear to be influenced by multiple factors, including molecular design, therapeutic mechanism, and clinical comparison context. Future studies incorporating biomarker-driven selection and mechanism-specific evaluation are needed to better define populations most likely to derive benefit from individual BsAb platforms.

Overall, BsAb-based regimens showed a generally manageable safety profile compared with non-BsAb antitumor therapies, although several mechanism-associated toxicity signals warrant attention. Renal and vascular toxicities represented the most prominent safety signals in our analysis. Proteinuria, hypertension, and peripheral edema were all significantly increased in the BsAb group, with consistent findings observed for grade ≥3 events. Importantly, these toxicities are unlikely to represent a universal adverse effect of all BsAbs but may be associated with specific molecular designs, particularly those involving VEGF pathway inhibition (36). VEGF signaling plays an essential role in maintaining glomerular endothelial function and podocyte homeostasis (56). VEGF blockade has been well recognized to disrupt glomerular endothelial integrity, leading to increased protein leakage and subsequent proteinuria (57). Blood pressure monitoring, antihypertensive management, and early assessment of urinary protein levels may help mitigate VEGF inhibitor-associated vascular and renal complications. Angiotensin-converting enzyme inhibitors or angiotensin receptor blockers may be considered in patients with hypertension accompanied by proteinuria, while severe renal toxicity or uncontrolled hypertension may require treatment interruption (58). Immune-related and inflammatory toxicities also require attention. In our analysis, CRS and pyrexia were more frequently observed in the BsAb group, although severe CRS and pyrexia did not significantly differ between groups. This pattern is consistent with the mechanism of CD3-engaging BsAbs, in which immune-cell recruitment and rapid cytokine release after immune synapse formation may result in inflammatory toxicities (59, 60). In addition, grade ≥3 rash was increased in the BsAb group. Although the prognostic significance of immune-related cutaneous events has mainly been established in ICI therapy, previous meta-analyses have suggested that immune-related cutaneous toxicities, including rash, may correlate with improved response and survival outcomes (61). Whether similar associations exist in BsAb-treated populations remains unclear and requires further investigation. In terms of gastrointestinal adverse events, all-grade decreased appetite and vomiting occurred more frequent in the BsAb group. These events were generally low grade and manageable with supportive care, appropriate dose adjustments, and routine clinical monitoring. The findings for hematologic toxicity were less uniform. All-grade leukopenia was reported less often in the BsAb group, whereas grade ≥3 anemia was more frequent. Given that several included trials used BsAb-containing combination regimens, this pattern may be influenced by concomitant chemotherapy, baseline disease burden, or disease-related bone marrow suppression, rather than representing a clear class-specific toxicity of BsAbs. Importantly, neutropenia did not show a consistent increase across analyses. Among other adverse events, pain in extremity was increased in the BsAb group and should be monitored in clinical practice.

This study has several strengths. To our knowledge, this is the first meta-analysis to comprehensively evaluate the efficacy and safety of BsAbs compared with non-BsAb antitumor therapies in solid tumors using randomized controlled trials. By focusing on active-comparator designs rather than placebo-controlled settings, our analysis provides a more clinically relevant assessment of the incremental value of BsAbs. However, several limitations should be acknowledged. First, the number of available randomized trials was relatively limited, reflecting the emerging clinical development stage of BsAbs in solid tumors. In addition, several subgroup analyses were based on a small number of studies and should therefore be interpreted as exploratory. Second, substantial heterogeneity was observed across studies, which was expected given the diversity of BsAb platforms, including differences in molecular mechanisms, target selection, tumor types, and comparator regimens. To address this issue, random-effects models, subgroup analyses, meta-regression, and sensitivity analyses were performed. However, residual heterogeneity could not be completely eliminated due to the inherent biological and clinical complexity of BsAbs. Therefore, the pooled estimates should be interpreted as an overall effect of currently available BsAb strategies rather than a universal class effect across all BsAb platforms. Third, some mechanism-specific safety outcomes, such as certain immune-related adverse events (e.g., hepatitis) and infections, could not be quantitatively synthesized because they were inconsistently reported across trials. Fourth, publication-bias analyses should be interpreted cautiously because only nine studies were included, limiting the power to detect potential asymmetry. Finally, although the Yang JC, 2025 trial evaluated a BsAb-containing combination regimen, sensitivity analysis excluding this study showed consistent results, suggesting that the overall efficacy findings were not substantially influenced by this treatment strategy.

## 5 Conclusion

BsAbs demonstrated improvements in PFS and OS compared with non-BsAb antitumor therapies in patients with solid tumors, while showing a generally manageable safety profile. Subgroup analyses further suggested that the survival benefit may be more pronounced in patients treated with T-cell engaging BsAbs or tumor microenvironment/angiogenesis-modulating BsAbs, patients aged <65 years, and patients with NSCLC. However, renal and vascular toxicities, as well as immune-related/inflammatory toxicities, warrant careful clinical attention. Further large-scale randomized trials are warranted to refine patient selection.

## Data Availability

The original contributions presented in the study are included in the article/**Supplementary Material**. Further inquiries can be directed to the corresponding authors.
