# Supplementary material for: Efficacy and safety of bispecific antibodies versus other antitumor therapies in solid tumors: a systematic review and meta-analysis
Source: Front Immunol. 2026 Aug 31;17:1931637. doi: 10.3389/fimmu.2026.1931637 (PMC13539596; doi:10.3389/fimmu.2026.1931637)

Supplementary Online Content

Efficacy and Safety of Bispecific Antibodies versus Other Antitumor Therapies in Solid Tumors: A Systematic Review and Meta-analysis

Table S1. Detailed Search Strategy

Table S2. Characteristics of included articles

Table S3. Exploratory subgroup analysis of PFS and OS

Table S4. Quality assessment of the evidence of the included publications, based on the GRADE

Table S5. Subgroup and meta-regression analysis for ORR

Figure S1. Risk of Bias Graph: Reviews of Authors' Judgments about Each Risk of Bias Item Are Presented as Percentages Across all Included Studies

Figure S2. Risk of Bias Summary: Reviews of Authors' Judgments about Each Risk of Bias Item for Each Included Study

Figure S3. Funnel Plot of PFS

Figure S4. Funnel Plot of OS

Figure S5. Funnel Plot of ORR

Table S1. Detailed Search Strategy

Data searched from PubMed

Search Query	Results
#1 "Antibodies, Bispecific"[Mesh] OR Bispecific Antibodies OR Bispecific Antibody OR Bispecific Monoclonal Antibodies OR Bifunctional Antibodies OR Catumaxomab OR Removab OR blinatumomab OR Blincyto OR amivantamab OR Rybrevant OR tebentafusp OR Kimmtrak OR mosunetuzumab OR BTCT-4465A OR Lunsumio OR cadonilimab OR AK104 OR teclistamab OR Tecvayli OR glofitamab OR Columvi OR talquetamab OR Talvey OR elranatamab OR Elrexfio OR epcoritamab OR Epkinly OR Odronextamab OR REGN1979 OR Linvoseltamab OR REGN5458 OR tarlatamab OR Imdelltra OR AMG 757 OR Erfonrilimab OR KN-046 OR Vudalimab OR XmAb20717 OR Volrustomig OR MEDI5752 OR Rilvegostomig OR AZD2936 OR Tebotelimab OR MGD013 OR Retlirafusp alfa OR SHR-1701 OR PM8002 OR ivonescimab OR AK112 OR Izalontamab OR SI-B001 OR CTX-009 OR Zenocutuzumab OR MCLA-128 OR Zanidatamab OR ZW25 OR Anbenitamab OR KN026	14,134
#2 "Neoplasms"[Mesh] OR neoplas* OR tumor* OR cancer* OR malignan* OR tumour* OR carcinom* OR adenocarcin*	6,522,629
#3 ((((((((randomized controlled trial[Publication Type]) OR controlled clinical trial[Publication Type]) OR randomized[Title/Abstract]) OR placebo[Title/Abstract]) OR randomly[Title/Abstract]) OR "Clinical Trials as Topic"[Mesh:NoExp]) OR trial[Title])) NOT ((((animals[MeSH Terms]) NOT ((humans[MeSH Terms]) AND animals[MeSH Terms])))))))	1,688,996
#4 #1 AND #2 AND #3	731

Searched before August 2026. Results: 731.

Data searched from Embase

Search Query	Results
#1 'bispecific antibody'/exp OR 'bispecific antibody' OR 'catumaxomab' OR 'blinatumomab' OR 'amivantamab' OR 'tebentafusp' OR 'mosunetuzumab' OR 'cadonilimab' OR 'teclistamab' OR 'glofitamab' OR 'talquetamab' OR 'elranatamab' OR 'epcoritamab' OR 'odronextamab' OR 'linvoseltamab' OR 'tarlatamab' OR 'erfonrilimab' OR 'vudalimab' OR 'volrustomig' OR 'rilvegostomig' OR 'tebotelimab' OR 'retlirafusp alfa' OR 'ivonescimab' OR 'izalontamab' OR CTX-009 OR 'zenocutuzumab' OR 'zanidatamab' OR 'anbenitamab'	26,025
#2 'neoplasm'/exp OR neoplas* OR tumor* OR cancer* OR malignan* OR tumour* OR carcinom* OR adenocarcin*	9,705,541
#3 'crossover procedure':de OR 'double-blind procedure':de OR 'randomized controlled trial':de OR 'single-blind procedured':de OR (random* OR factorial* OR crossover* OR cross NEXT/1 over* OR placebo* OR doubl* NEAR/1 blind* OR singl* NEAR/1 blind* OR assign* OR allocat* OR volunteer*):de,ab,ti	4,218,787

#4 #1 AND #2 AND #3	3,334

Searched before August 2026. Results: 3,334.

Data searched from Cochrane Library

Search Query	Results
#1 MeSH descriptor: [Antibodies, Bispecific] explode all trees	166
#2 (catumaxomab):ti,ab,kw OR (blinatumomab):ti,ab,kw OR (amivantamab):ti,ab,kw OR (tebentafusp):ti,ab,kw OR (mosunetuzumab):ti,ab,kw OR (cadonilimab):ti,ab,kw OR (teclistamab):ti,ab,kw OR (glofitamab):ti,ab,kw OR (talquetamab):ti,ab,kw OR (elranatamab):ti,ab,kw OR (epcoritamab):ti,ab,kw OR (odronextamab):ti,ab,kw OR (linvoseltamab):ti,ab,kw OR (tarlatamab):ti,ab,kw OR (erfonrilimab):ti,ab,kw OR (vudalimab):ti,ab,kw OR (volrustomig):ti,ab,kw OR (rilvegostomig):ti,ab,kw OR (tebotelimab):ti,ab,kw OR (retlirafusp alfa):ti,ab,kw OR (ivonescimab):ti,ab,kw OR (izalontamab):ti,ab,kw OR (CTX-009):ti,ab,kw OR (zenocutuzumab):ti,ab,kw OR (zanidatamab):ti,ab,kw OR (anbenitamab):ti,ab,kw	974
#3 MeSH descriptor: [Neoplasms] explode all trees	135,785
#4 (cancer):ti,ab,kw OR (tumor):ti,ab,kw OR (adenocarcinoma):ti,ab,kw	268,414
#5 #1 OR #2	1181
#6 #3 OR #4	310,725
#7 #5 AND #6	920

Searched before August 2026. Results: 920.

Data searched from Scopus

Search Query	Results
#1 TITLE-ABS-KEY (random* OR placebo* OR "controlled trial" OR "randomized controlled trial" OR "randomised controlled trial" OR "clinical trial" OR "crossover" OR "double-blind" OR "single-blind")	5,494,524
#2 TITLE-ABS-KEY (neoplas* OR tumor* OR cancer* OR malignan* OR tumour* OR carcinom* OR adenocarcin*)	7,357,698
#3 TITLE-ABS-KEY ("Antibodies, Bispecific" OR "Bispecific Antibodies" OR "Bispecific Antibody" OR "Bispecific Monoclonal Antibodies" OR "Bifunctional Antibodies" OR Catumaxomab OR Removab OR blinatumomab OR Blincyto OR amivantamab OR Rybrevant OR tebentafusp OR Kimmtrak OR mosunetuzumab OR BTCT-4465A OR Lunsumio OR cadonilimab OR AK104 OR teclistamab OR Tecvayli OR glofitamab OR Columvi OR talquetamab OR Talvey OR elranatamab OR Elrexfio OR epcoritamab OR EpkinlyOR Odronextamab OR REGN1979 OR Linvoseltamab OR REGN5458 OR tarlatamab OR Imdelltra OR "AMG 757" OR Erfonrilimab OR KN-046 OR Vudalimab OR XmAb20717 OR Volrustomig OR MEDI5752 OR Rilve	16,743

gostomig OR AZD2936 OR Tebotelimab OR MGD013 OR "Retlirafusp alfa" OR SHR-1701 OR PM8002 OR ivonescimab OR AK112 OR Izalontamab OR SI-B001 OR CTX-009 OR Zenocutuzumab OR MCLA-128 OR Zanidatamab OR ZW25 OR Anbenitamab OR KN026)	
#4 #1 AND #2 AND #3	4,519

Searched before August 2026. Results: 4,519.

Table S2. Characteristics of included articles

Author, year	bsAb formats	Masking	Phase	No. of male	Target
Bendell JC, 2020	IgG-like	double-blind	2	94	Ang2×VEGF
Chen Z, 2025	IgG-like	double-blind	3	494	PD1×VEGF-A
Fayette J, 2016	IgG-like	open-label	2	101	EGFR×HER3
Hassel JC, 2023	Non-IgG-like	open-label	3	190	gp100×CD3
Hill AG, 2018	IgG-like	open-label	2	82	EGFR×HER3
Mountzios G, 2025	Non-IgG-like	open-label	3	351	DLL3×CD3
Xiong A, 2025	IgG-like	double-blind	3	333	PD1×VEGF-A
Yang JC, 2025	IgG-like	NA	3	332	EGFR×MET
Yang Y, 2025	IgG-like	open-label	3	321	EGFR×HER3

bsAb, bispecific antibody; NA, not Available; IgG, Immunoglobulin G; Ang, Angiopoietin-2; VEGF, Vascular Endothelial Growth Factor; PD-L1, programmed cell death-ligand 1; EGFR, Epidermal Growth Factor Receptor; HER3, Human Epidermal Growth Factor Receptor 3; gp100, Glycoprotein 100; CD3, Cluster of Differentiation 3; DLL3, Delta-Like Protein 3; MET, Mesenchymal Epithelial Transition Factor.

Table S3. Exploratory subgroup analysis of PFS and OS.

Subgroup	PFS				OS			
	No. of studies	HR (95% CI)	I^2 %	p	No. of studies	HR (95% CI)	I^2 %	p
Age								
<65 yrs	3	0.56 (0.37, 0.84)	72.8	0.01	3	0.56 (0.46, 0.67)	0	<0.01
≥65 yrs	3	0.67 (0.52, 0.88)	43.7	<0.01	2	0.86 (0.64, 1.16)	61.4	0.34
Brain metastasis								
Yes	2	0.59 (0.45, 0.77)	0.0	<0.01	2	0.56 (0.38, 0.82)	63.3	<0.01
No	3	0.68 (0.52, 0.89)	74.0	<0.01	2	0.82 (0.66, 1.01)	0	0.01
ECOG PS								
0	3	0.35 (0.18, 0.68)	54.7	<0.01	NA	NA	NA	NA
1	3	0.57 (0.48, 0.69)	0.0	<0.01	NA	NA	NA	NA
Liver metastasis								
Yes	4	0.61 (0.44, 0.85)	42.6	<0.01	NA	NA	NA	NA
No	4	0.57 (0.45, 0.72)	52.0	<0.01	NA	NA	NA	NA
PD-L1								
1-49%	2	0.56 (0.40, 0.78)	0	<0.01	NA	NA	NA	NA
≥50%	2	0.58 (0.42, 0.80)	0	<0.01	NA	NA	NA	NA
Race								
Asian	NA	NA	NA	NA	3	0.63 (0.43, 0.91)	70.2	0.02
Non-Asian	NA	NA	NA	NA	2	0.74 (0.58, 0.95)	0.0	0.02
Sex								
Female	3	0.55 (0.41, 0.75)	0.0	<0.01	3	0.65 (0.50, 0.86)	43.7	<0.01
Male	4	0.58 (0.46, 0.74)	63.5	<0.01	3	0.73 (0.61, 0.87)	0.0	<0.01

yrs, years; ECOG PS, Eastern Cooperative Oncology Group Performance Status; PD-L1, Programmed cell death-Ligand 1; No, number; PFS, progression-free survival; HR, hazard ratio; OS, overall survival; NA, not available.

Table S4. Quality assessment of the evidence of the included publications, based on the GRADE.

Quality assessment							No of patients		Effect		Quality	Importance
**No of studies**	**Design**	**Risk of bias**	**Inconsistency**	**Indirectness**	**Imprecision**	**Other considerations**	**Intervention**	**Control**	**Relative (95% CI)**	**Absolute**		
**PFS**												
9	RCT	not serious	not serious	serious	not serious	none	1811	1694	HR:0.76 (0.61,0.94)	-- per 1,000 (from -- to --)[a]	⊕⊕⊕O MODERATE	CRITICAL
**OS**												
5	RCT	not serious	not serious	serious	not serious	none	824	704	HR:0.78 (0.63,0.95)	-- per 1,000 (from -- to --)[b]	⊕⊕⊕O MODERATE	CRITICAL
**ORR**												
9	RCT	not serious	not serious	serious	serious	none	1739	1614	RR:1.20 (1.00,1.44)	94 more per 1,000 (from 0 fewer to 207 more)	⊕⊕OO LOW	IMPORTANT

No, numbers; CI, confidence interval; PFS, progress-free survival; RCT, randomized controlled trial; HR, hazard ratio; RR, relative risk.

a. Absolute effect estimates could not be calculated for progression-free survival, because aggregated event numbers across included trials were not available; only hazard ratio was reported.

b. Absolute effect estimates could not be calculated for overall survival, as aggregated event numbers across included trials were unavailable; only hazard ratio was pooled for analysis.

Table S5. Subgroup and meta-regression analysis for ORR

Subgroup	No. of studies	RR (95% CI)	I^2 %	p	p for meta-regression
BsAb format					
Non-IgG-like	2	1.77 (1.34, 2.35)	0.0	<0.001	0.248
IgG-like	7	1.11 (0.94, 1.32)	78.2	0.218	
BsAbs mechanism					
T-cell engaging BsAbs	2	1.77 (1.34, 2.35)	0.0	<0.001	0.275
Tumor microenvironment-modulating BsAbs	3	1.12 (0.95, 1.32)	55.4	0.185	
Tumor signaling pathway-targetin BsAbs	4	1.03 (0.62, 1.71)	85.4	0.902	
Cancer type					
CRC	2	0.73 (0.42, 1.27)	63.8	0.268	0.157
NSCLC	4	1.22 (1.02, 1.45)	84.1	0.033	
Comparator therapy					
Chemotherapy	2	1.84 (1.47, 2.30)	0.0	<0.001	0.002
Immunotherapy	3	1.24 (1.02, 1.52)	50.5	0.035	
Targeted therapy	4	0.91 (0.73, 1.13)	48.5	0.371	

CRC, colorectal Cancer; NSCLC, non-small cell lung cancer; No, number; RR, relative risk; NA, not available.

Figure S1. Risk of Bias Graph: Reviews of Authors' Judgments About Each Risk of Bias Item Are Presented as Percentages Across all Included Studies

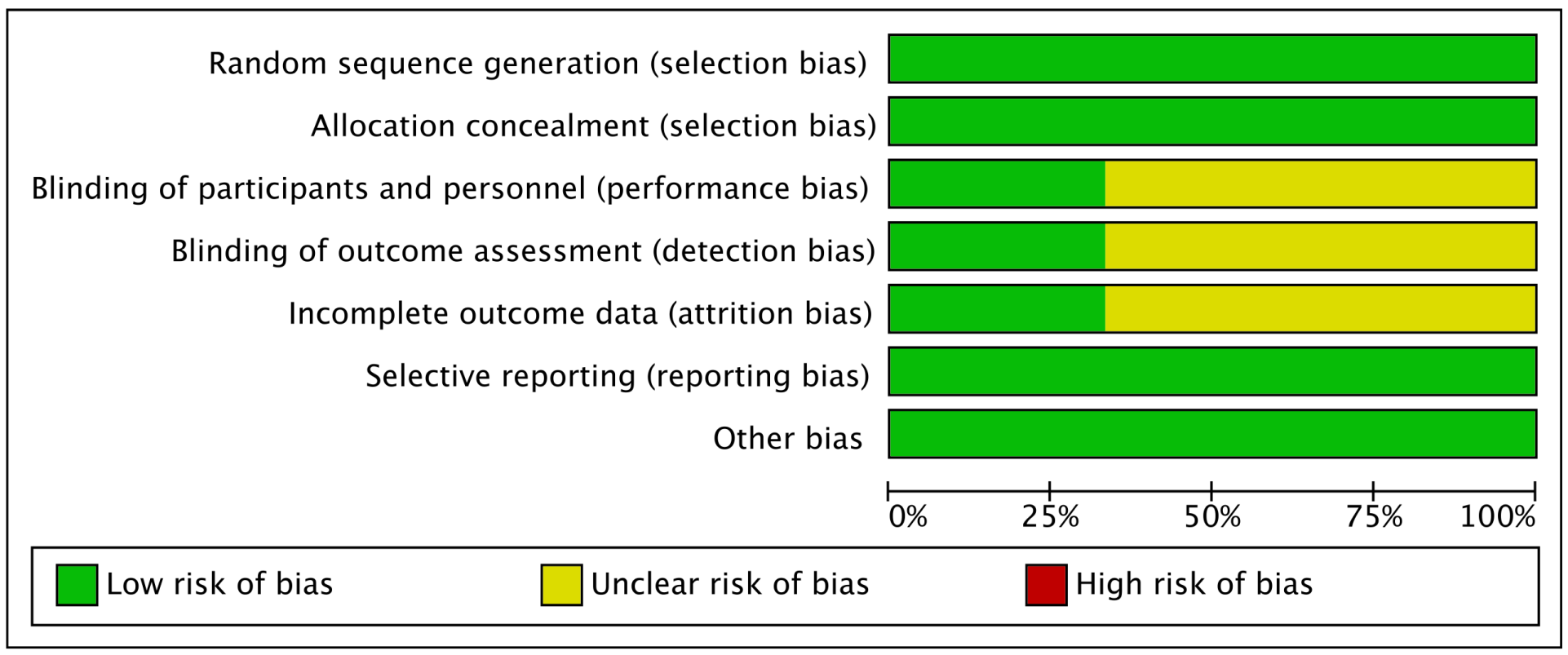

Figure S2. Risk of Bias Summary: Reviews of Authors' Judgments About Each Risk of Bias Item for Each Included Study

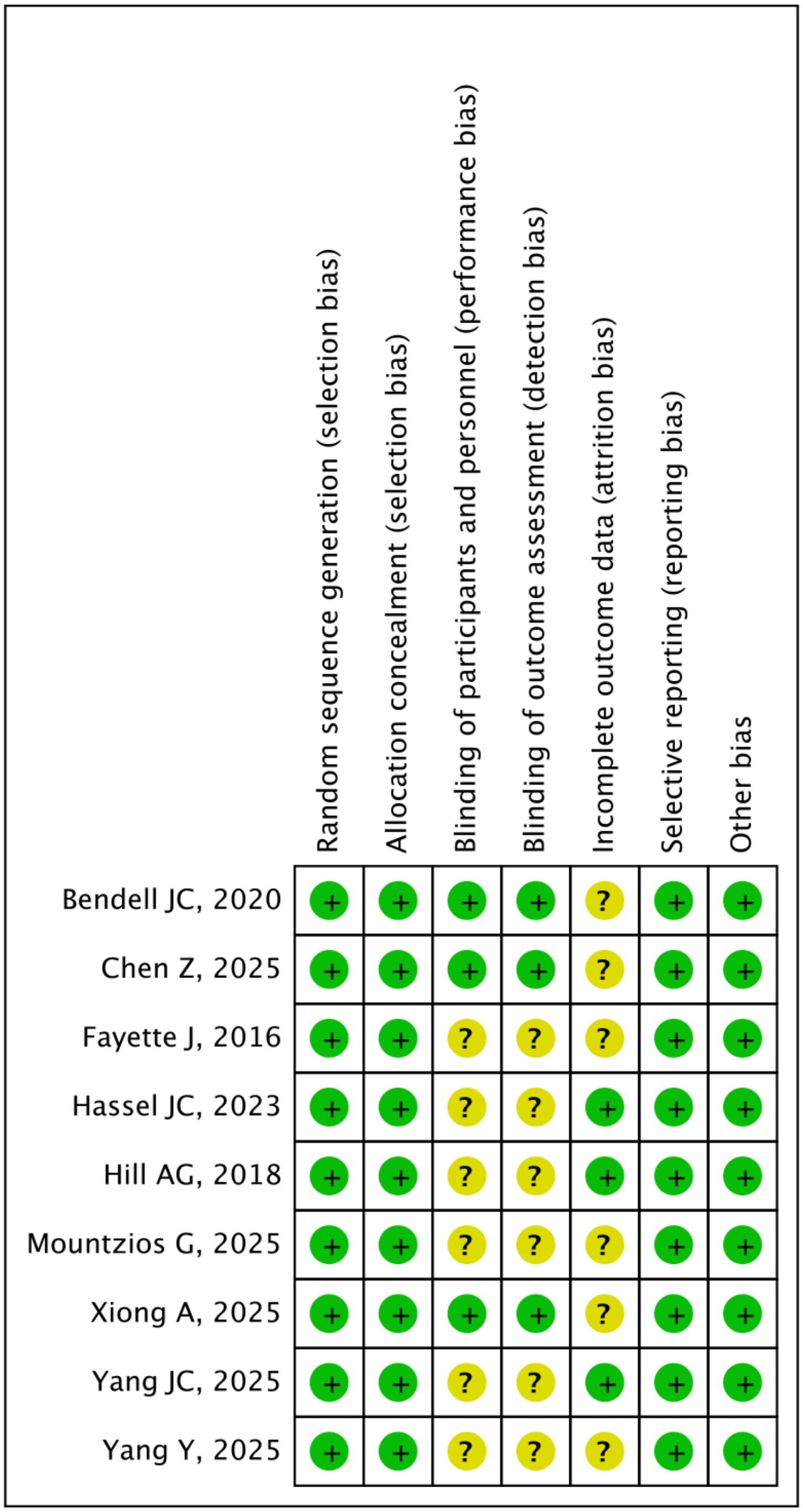

Figure S3. Funnel Plot of PFS

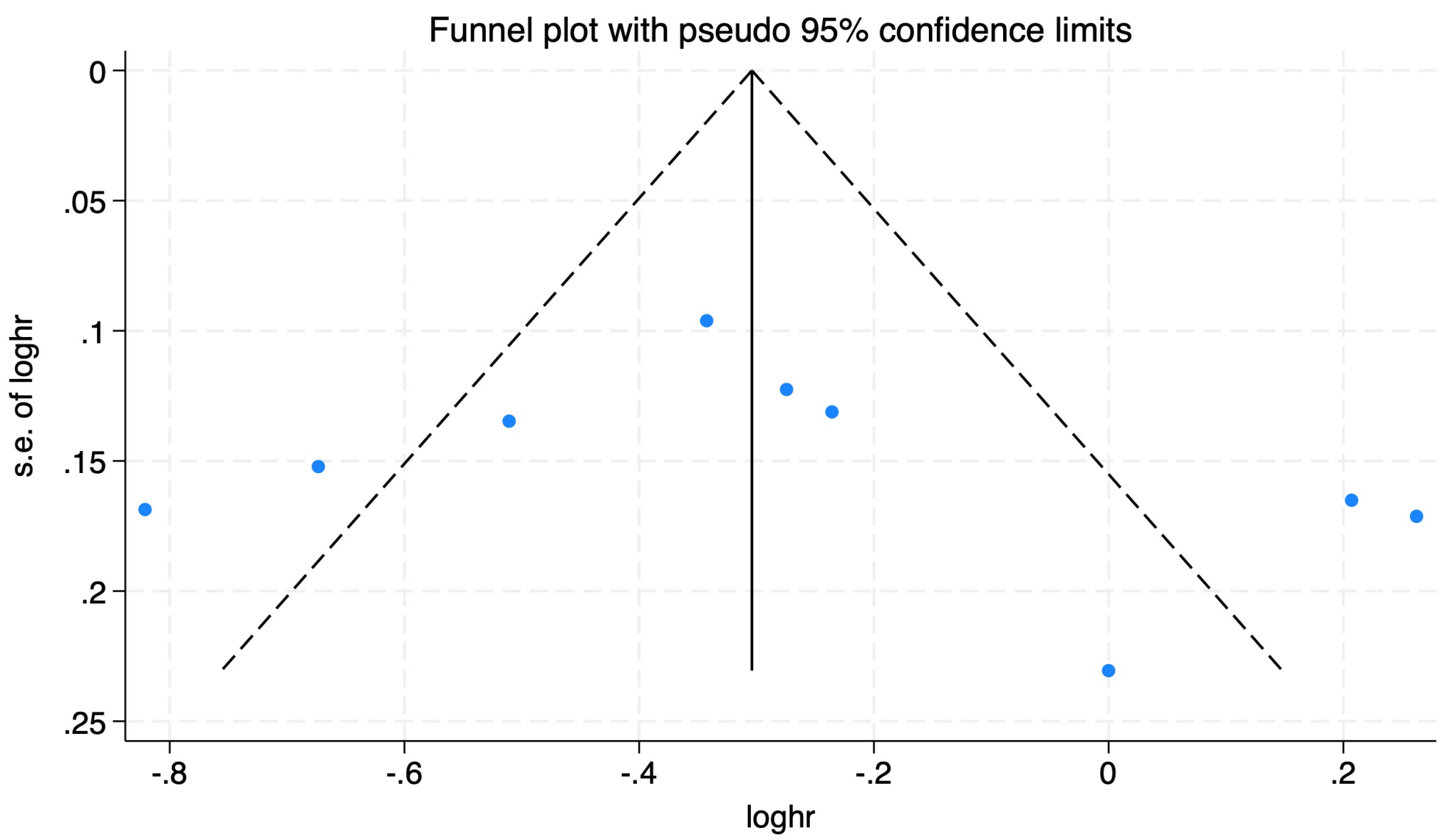

Figure S4. Funnel Plot of OS

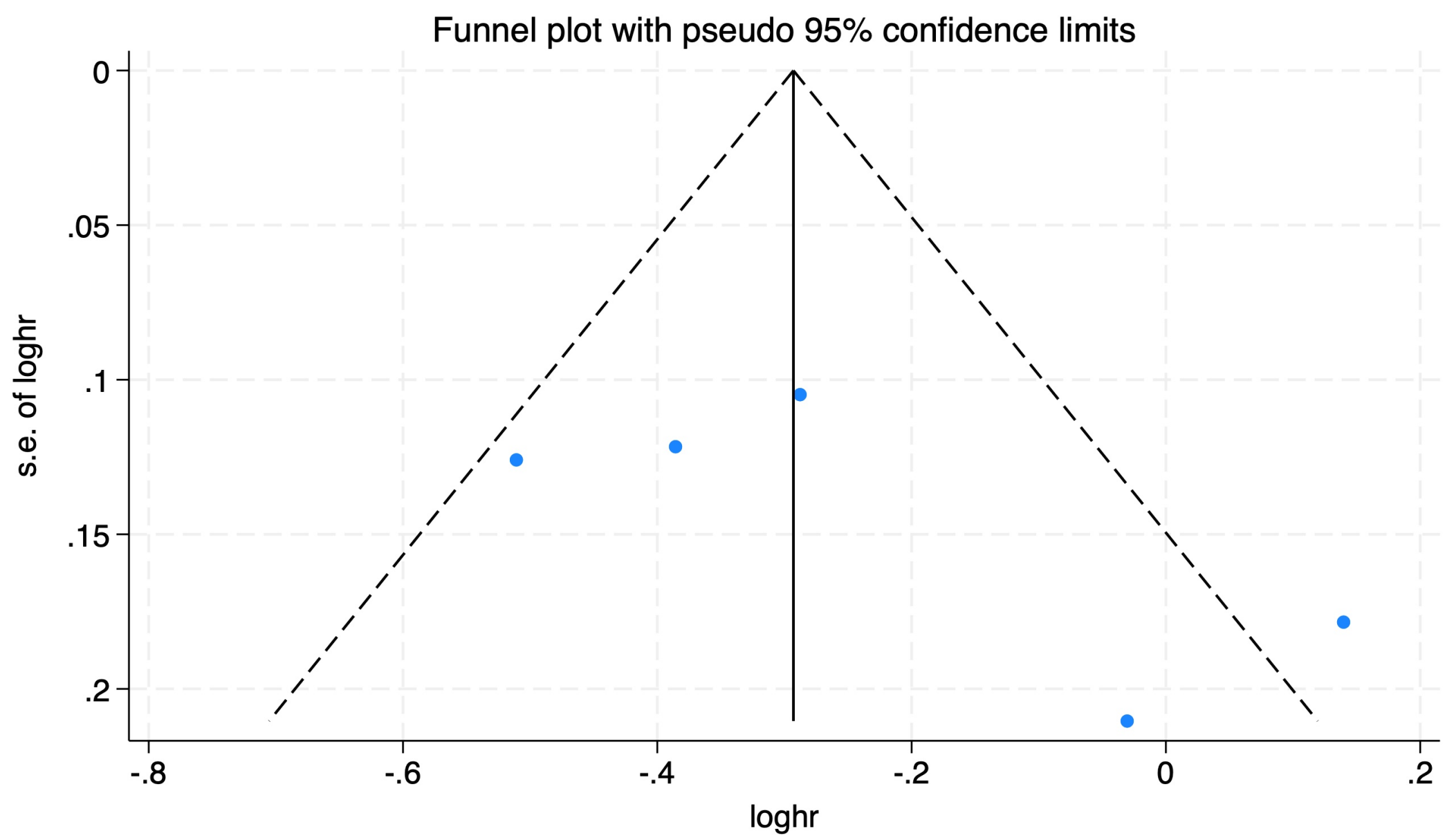

Figure S5. Funnel Plot of ORR

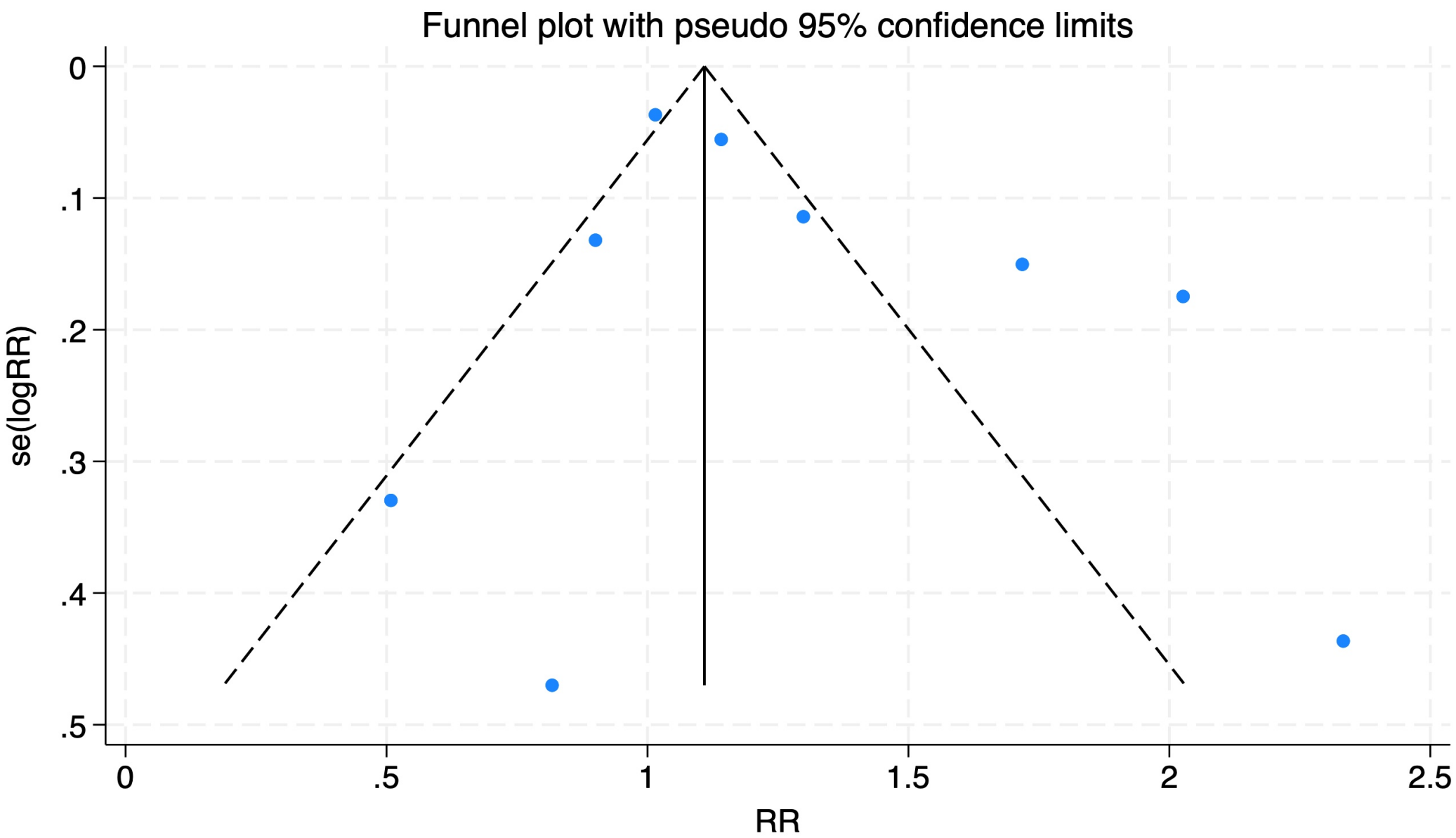

Supplement: Supplementary file 1 [file DataSheet1.pdf]
